# Delirium in Home Care: A Case Report

**DOI:** 10.7759/cureus.47094

**Published:** 2023-10-16

**Authors:** Kelly Sabbe, Roos Van Der Mast, Bart Van Rompaey

**Affiliations:** 1 Medicine and Health Sciences, University of Antwerp, Antwerp, BEL; 2 Psychiatry and Behavioral Sciences, University of Leiden, Leiden, NLD; 3 Family Medicine and Population Health, University of Antwerp, Antwerp, BEL

**Keywords:** infection induced delirium, persistent delirium, community health nurse (c.h.n.), family caregiver, primary caregiver, general practice < general medicine, home health care, mixed delirium

## Abstract

Delirium is a challenging medical problem, particularly in the home care setting, and greatly affects both patients and family caregivers. When delirium is not immediately detected and effectively managed, various outcomes are adversely affected. This report describes delirium in an older home-bound man and offers strategies for detecting and managing delirium in a home care setting.

The patient is a frail 86-year-old man with multiple medical comorbidities and functional decline after bronchitis that was diagnosed by a general practitioner. Following the diagnosis and subsequent treatment of bronchitis, the patient suffered a major decline in cognitive and physical functioning during normal daily activities.

Medical screening revealed confusion, apathy, and extreme fatigue. Using the assessment tool of the Functional Independence Measures and Delirium Observation Screening Scale (DOSS), the presence of functional decline and delirious symptoms were found.

Through multidisciplinary collaboration, a treatment plan was initiated. It consisted of hydration following a fixed schedule, adapted nutrition, a temporary adapted medication schedule for pre-diabetes, and an exercise plan. No specific pharmaceuticals were given. The patient made a full recovery over time.

All professional and informal caregivers should be aware of the potential presence of delirium when an older patient with a deteriorating physical or mental condition presents itself. Good diagnostics for delirium and possible underlying diseases are necessary. Adequate treatment, with the help of paramedics such as dieticians, physiotherapists, etc. must be provided when necessary.

## Introduction

Delirium is by definition a disturbance in attention and awareness developing over a short period of time and represents an acute change in attention and awareness [1]. The condition tends to fluctuate in severity during the course of a day [1]. Delirium is common among hospitalized older patients. The prevalence of delirium at admission to the general hospital ranges from 10% to 31%, suggesting that delirium often develops before hospitalization (or at admission) and may be an important reason for hospitalization [2]. Delirium is a challenging and under-diagnosed medical problem in the home care setting and greatly affects both patients and family caregivers [3,4]. When delirium is not detected early and managed effectively, outcome and quality of life are adversely affected [3].

Delirium is particularly common in older patients with medical multimorbidity and may develop from exacerbations of a chronic illness, the onset of an acute illness, or a medication side effect [2,4]. Importantly, delirium may also develop from a rather benign medical disturbance, particularly in high-risk patients [4,5]. Therefore, these high-risk patients require close observation since they are susceptible to the development of delirium and need prompt intervention in case of delirium.

It is unclear whether, in a home care setting, adequate attention is being paid to the development of delirium in frail home-bound patients [4]. Informal caregivers, home healthcare nurses, and general practitioners play a key role in the early identification, assessment, and management of delirium [6]. Furthermore, home healthcare nurses and general practitioners play a crucial role in the support and education of patients and their informal caregivers [2,3,5]. The physical examination of older patients can present great challenges to general practitioners and home care nurses, especially if the patients are uncooperative (due to delirium). It should be stressed that lack of cooperation for unknown reasons can be an important indication of delirium due to various medical disturbances [5].

To illustrate delirium in a home care setting, this case report of a home-bound older man describes the observations, assessments, and interventions for delirium caused by acute illness. Careful and targeted diagnostics are required to assist the home care nurse and the general practitioner in the decision-making process of treatment planning. Declines in functional and cognitive abilities and changes in clinical indicators are being used to determine the course of action.

## Case presentation

The patient was a frail 86-year-old man with multiple medical comorbidities including obesity, hypertension, pre-diabetes, and polypharmacy (see Table 1 part A) who showed sudden clinical deterioration, fever, and a dry cough. An appointment with the general practitioner was made. After clinical examination, the patient was diagnosed with bronchitis by the general practitioner. A blood sample was taken to confirm the diagnosis. No mental deterioration was present.

**Table 1:** Medication regimen All medication doses are in mg.

Name	8h00	12h00	16h00	18h00	20h00	Problem
A. Regular medication						
Novonorm	1			1		Pre-diabetes
Glucophage	850					Pre-diabetes
Forzaten/HCT	40	5		12		High blood pressure
Bisoprolol	2.5					Elevated heart rate
Eliquis	5			5		Blood clots
Atorvastatin	20					High cholesterol
Allopurinol	300					Uric acid
B. Medication to treat the bronchitis						
Inuvair Nexthaler	1	1		1		Mucus
Lysotossil syrup	15	15		30		Dry cough
C. Antibiotics to treat the pharyngitis						
Amoxicillin	500			500		General antibiotics
D. Additional medication for cataract procedures						
Mydriasert		0.28/5.4				Diluting eye, day of procedure
Indocollyre	1 drop	1 drop	1 drop		1 drop	3 days before, and until 4 weeks after the procedure
De Icol	1 drop	1 drop	1 drop		1 drop	From day 1 until day 3 after operation
Paracetamol	500	500	500		500	Pain, when needed

The blood sample showed increased CRP values, an inflammation marker, ranging from 100 mg/l to >300 mg/l. No other values were deviant from normal. No other screenings were performed. The patient refused urine analyses and additional blood samples. In the following two weeks, the patient experienced multiple febrile episodes. The prescribed bronchitis treatment consisted of Inuvair Nexthaler 1 inhalation three times per day for two weeks (see Table 1 part B). In the second week, the general practitioner prescribed additional Lysotossil syrup 15 ml in the morning and at midday and Lysotossil 30 mg before bedtime for the dry cough that persisted (see Table 1 part B). Few other symptoms were present, such as a loss of appetite and fatigue.

Since the patient showed no clinical improvement after two weeks, the general practitioner did a follow-up consultation, where acute pharyngitis was diagnosed upon physical examination. Amoxicillin 500 mg two times a day for 10 days was prescribed to treat the pharyngitis, whereafter the patient seemed to recover gradually (see Table 1 part C). Although he initially recovered from the bronchitis, the patient showed an obvious functional and cognitive decline upon resuming his daily activities in the second week. He was no longer able to perform a range of activities of daily living (ADL), going as far as stopping eating and drinking independently (see Table 2). He became febrile again and appeared delirious, which was noticed by the home care nurse.

**Table 2:** Katz-ADL assessment ADL: activities of daily living

Katz index of independence in ADL		
Activities	Independence (1 point)	Dependence (0 points)
Points (1 or 0)	NO supervision, direction, or personal assistance.	WITH supervision, direction, personal assistance, or total care.
Bathing	Bathes self completely or needs help in bathing only a single part of the body such as the back, genital area, or disabled extremity.	Need help with bathing more than one part of the body, getting in or out of the tub or shower. Requires total bathing.
Points: 0		
Dressing	Get clothes from closets and drawers and put on clothes and outer garments complete with fasteners. May need help tying shoes.	Needs help with dressing self or needs to be completely dressed.
Points: 0		
Toileting	Goes to the toilet, gets on and off, arranges clothes, and cleans the genital area without help.	Needs help transferring to the toilet, cleaning self, or using a bedpan or commode.
Points: 1		
Transferring	Moves in and out of bed or chair unassisted. Mechanical transfer aids are acceptable.	Needs help in moving from bed to chair or requires a complete transfer.
Points: 1		
Continence	Exercises complete self-control over urination and defecation.	Is partially or totally incontinent of bowel or bladder.
Points: 1		
Feeding	Gets food from a plate into the mouth without help. Preparation of food may be done by another person.	Needs partial or total help with feeding or requires parenteral feeding.
Points: 0		
Total points: 3		
Scoring: 6 = High (patient independent) 0 = Low (patient very dependent)		

Investigations

The follow-up clinical examination by the general practitioner revealed mild dehydration. The comprehensive systems screen revealed increased pallor, loose and frequent bowel movements, confusion, apathy, and extreme fatigue. Assessment with the Katz-ADL showed a significant decline in functional independence from six points normally to three points (see Table 2) [7,8].

The results of the clinical examination were auditive hallucinations, restlessness, agitation, combative behavior, withdrawal, slowed movement, disturbed sleep habits, impaired orientation, and speech and temporal fluctuation, which were consistent with indicators for the presence of delirium, dehydration, and malnutrition [9]. These findings were reported to the patient's family members by the home care nurse. A treatment plan was developed in collaboration with the general practitioner, home care nurse, and family caregivers and carried out.

For the required daily delirium screening, the help of the family caregivers was enlisted. The home care nurse taught them the use of the Single Question in Delirium (SQiD): "Do you think [patient] has been more confused lately?" [10]. If the family felt that the patient was more confused than normal, the home care nurse was called upon which she screened the patient with the Delirium Observation Screening Scale (DOSS) (see Table 3) [11]. The results of the initial delirium screening performed by the healthcare nurse showed a score of 6/13. When there was a positive screening result with the DOSS, the general practitioner was notified to discuss further possible interventions.

**Table 3:** Detailed results of the initial screening with the DOSS (Day 3)

Patient observation		Morning			Noon			Evening		
		Never	Sometimes - always	Unable	Never	Sometimes – always	Unable	Never	Sometimes - always	Unable
1	Dozes off during conversation or activities	0	1 X	-	0	1 X	-	0 X	1	-
2	Easily distracted by stimuli from the environment	0	1 X	-	0	1 X	-	0	1 X	-
3	Maintains attention to conversation or action	1 X	0	-	1 X	0	-	1 X	0	-
4	Does not finish a question or answer	0 X	1	-	0 X	1	-	0 X	1	-
5	Gives answers that do not fit the question	0 X	1	-	0 X	1	-	0 X	1	-
6	Reacts slowly to instructions	0	1 X	-	0	1 X	-	0	1 X	-
7	Thinks to be somewhere else	0 X	1	-	0 X	1	-	0 X	1	-
8	Knows which part of the day it is	1	0 X	-	1	0 X	-	1	0 X	-
9	Remembers recent event	1 X	0	-	1 X	0	-	1	0 X	-
10	Is picking, disorderly, restless	0 X	1	-	0 X	1	-	0 X	1	-
11	Pulls IV tubes, feeding tubes, catheters, etc.	0 X	1	-	0 X	1	-	0 X	1	-
12	Is easy or sudden emotional	0 X	1	-	0 X	1	-	0 X	1	-
13	Sees/hears things that are not there	0	1 X	-	0 X	1	-	0 X	1	-
Total score (0-13)		6			5			3		

Treatment

The treatment plan for this frail older patient had multiple aims: counteract the dehydration and malnutrition, support the family caregivers with ADL care, and educate him about delirium by the home care nurse.

After consultation with a dietician, a fixed hydration schedule with 1.5 liters of water was given to counteract the dehydration. The patient needed to drink a glass of 250 ml of water every two to three hours, six times a day. An adapted nutrition scheme with soft digestible, protein-rich food was also started to counteract the malnutrition and prevent obstipation. The general practitioner made a temporary adaptation to the medication schedule: a temporary Glucophage stop as long as the prescribed nutrition scheme was followed.

A care protocol was started with special attention to aspects that focus on delirium treatment and prevention of reoccurring delirium. These aspects consisted of continuous reorientation and support (calendars, clocks, pictures of family), using straightforward and concise communication. Special attention was given to the prevention of sensory overload or sensory deprivation by assessing and adapting the noise level and appropriateness of environmental stimuli, managing adequate and appropriate lighting around the clock, maintaining normal schedules and routines as much as possible, and providing sensory aids (glasses and magnifying glasses) as much as possible. Table 4 shows the schedule of events and delirium screening.

**Table 4:** Schedule of events and delirium screening ^1^DOSS: Delirium Observation Screening Scale, a 13-item observational screening scale. A score of ≥3 indicates the presence of delirium [8]. ^2^SQiD: Single Question in Delirium: "Do you think [patient] has been more confused lately?" [7].

Illness course	Event				
Before	Clinical deterioration, fever, and a dry cough were observed. An appointment with the general practitioner was made. No mental deterioration was present				
Day 1	Consultation by the general practitioner				
	Diagnosis: bronchitis				
	Prescription: Inuvair Nexthaler for 7 days				
Day 7	Follow-up consultation by the general practitioner				
	Diagnosis: bronchitis				
	Prescription: Inuvair Nexthaler and Lysotossil syrup for 7 days				
Day 7-14	Functional and cognitive declined, stopped eating and drinking independently, and appeared delirious according to the home care nurse. A general practitioner was called				
Day 14	Follow-up consultation by the general practitioner				
	Diagnosis: acute pharyngitis				
	Prescription: Amoxicillin for 10 days				
Illness course	Event	Screening^1, 2^	Morning	Midday	Evening
Day 14	Home healthcare nurse screened for delirium	DOSS	6	5	3
Day 15	Home healthcare nurse teaches family caregiver SQiD. When positive, additionally the patient was screened with the DOSS	SQiD	Yes	No	Yes
		DOSS	7		3
Day 16	Consultation with the dietician: start a hydration schedule and an adapted nutrition scheme with soft digestible, protein-rich food				
	Follow-up consultation by the general practitioner: temporary stop Glucophage				
	SQiD was positive, and the patient was additionally screened with DOSS	SQiD	Yes	Yes	Yes
		DOSS	9	7	4
Day 17		SQiD	No	No	No
Day 18		SQiD	No	No	No
Day 19	SQiD was positive, and the patient was additionally screened with DOSS	SQiD	No	Yes	Yes
		DOSS		4	4
Day 20	SQiD was positive, and the patient was additionally screened with DOSS	SQiD	Yes	Yes	No
		DOSS	7	5	
Day 21		SQiD	No	No	No
Day 22		SQiD	No	No	No
Day 23	SQiD was positive, and the patient was additionally screened with DOSS	SQiD	No	Yes	No
		DOSS		4	
Day 24	Stop antibiotics	SQiD	No	No	No
Day 25	Follow-up consultation by the general practitioner				
	Diagnosis: no more acute illness, no more dehydration, and no more malnutrition				
	Prescription: continue with regular medication				
Week 6	Cataract operation on the left eye	SQiD	No	No	No
Week 6, day 1	Check-up 1	SQiD	No	No	No
Week 6, day 2	Check-up 2	SQiD	No	No	No
Week 8	Cataract operation on the right eye	SQiD	No	No	No
Week 8, day 1	Check-up 1	SQiD	No	No	No
Week 8, day 2	Check-up 2	SQiD	No	No	No

Outcome and follow-up

After the first two weeks of observation, the frequency of delirious episodes and the intensity of delirium symptoms declined. At certain times during the following weeks, the family caregivers had the impression that the patient’s confusion level was increasing again. When this happened, the SQiD was performed on the spot in the same temporal and spatial context. The observations were always mentioned to the home care nurse who performed a delirium screening with the DOSS, which always gave negative results.

About one month later, the patient underwent a cataract procedure in the right eye and three weeks later in the left eye. During these procedures, Mydriasert was given to dilute the pupil, and a topical anesthetic was used, which can be a precipitant factor for delirium [12]. Therefore, the family caregivers were asked to evaluate the condition of the patient at least three times a day for a relapse of delirium using the SQiD [10]. The patient didn’t have a relapse, although the family caregivers described that the patient had possibly short "flashbacks" of the delirious episodes.

## Discussion

The results of the clinical examination were consistent with indicators for the presence of delirium, dehydration, and malnutrition. Although the general practitioner had a strict treatment protocol for the dehydration and malnutrition, there was no treatment protocol initially for the observed delirium. The home care nurse recognized the presence of delirium and the fact that dehydration and malnutrition are relevant precipitating factors for the current delirium [6,13].

Before the manifestation of the bronchitis and delirium, the patient had been very active with no cognitive impairment whatsoever, according to the records of the general practitioner. However, there were some vulnerabilities and high-risk factors present in the patients’ medical history for the development of delirium such as old age, decreased activity, vision impairment, obesity, underlying diabetes, polypharmacy, and hypertension.

The bronchitis infection in combination with dehydration and malnutrition was an obvious trigger for the development of delirium. Dehydration is a common risk factor for developing delirium and is one of the 10 most frequent diagnoses reported for hospitalizations in older people [14-16].

The symptoms of delirium can be frightening for informal caregivers who lack knowledge of delirium [3]. Delirium stays unrecognized in 33% to 66% of cases and has been mistaken for dementia, depression, mood disorders, and functional psychoses [9,13,17,18]. The cause of delirium is often multifactorial; two to six factors may be present in any single case.

At first, the family caregiver in our case attributed the confusion solely to bronchitis, as delirium was not well known. Learning from this case, routine cognitive testing, routine screening in laboratories for signs of infection, dehydration, and malnutrition, and the use of screening tools in-home care are suggested to improve delirium detection [18]. Furthermore, the literature suggests that patient outcomes are improved if training in risk factor recognition is performed [3,17,19,20].

This case report illustrates how knowledge of the pathologies associated with delirium and thorough examination can assist in making clinical decisions when home care patients require delirium management and care [4]. This highlights the need to appropriately manage patients at high risk of developing delirium, especially if suffering from a seemingly common infection.

## Conclusions

The best treatment for delirium in a home care setting is prevention. Hence, early recognition and management of risk factors for delirium are vital. A close collaboration between informal caregivers, nurses, and general practitioners not only helps in preventing delirium but also a deteriorating clinical condition and an increase in hospital admittance.

Important elements to improve the patient's cooperation include ensuring basic needs, sufficient time and patience, adequate communication, and good cooperation with informal and formal caregivers. Targeted clinical observation as well as thinking in geriatric syndromes and unmet needs can help to form a complete picture despite limited cooperation.
